# Exploring Outcomes to Consider in Economic Evaluations of Health Promotion Programs: What Broader Non-Health Outcomes Matter Most?

**DOI:** 10.1186/s12913-015-0908-y

**Published:** 2015-07-14

**Authors:** Tim M. Benning, Adrienne F.G. Alayli-Goebbels, Marie-Jeanne Aarts, Elly Stolk, G. Ardine de Wit, Rilana Prenger, Louise M.A. Braakman-Jansen, Silvia M.A.A. Evers

**Affiliations:** Caphri School of Public Health and Primary Care, Department of Health Services Research, Maastricht University, PO Box 616, Maastricht, 6200 MD The Netherlands; Institute of Health Economics and Clinical Epidemiology, University Hospital of Cologne, Gleueler Strasse 176-178, 50935, Koeln, Germany; Regional Public Health Service Limburg-Noord, PO Box 11, Venlo, 5900 AA The Netherlands; Institute of Health Policy & Management, Institute for Medical Technology Assessment, Erasmus University Rotterdam, PO Box 1738, Rotterdam, 3000 DR The Netherlands; Centre for Nutrition, Prevention and Healthcare, National Institute of Public Health and the Environment, PO Box 1, Bilthoven, 3720 BA The Netherlands; Julius Center for Health Sciences and Primary Care, University Medical Center Utrecht, PO Box 85500, Utrecht, 3508 GA The Netherlands; Department of Psychology, Health & Technology, University of Twente, PO Box 217, Enschede, 7500 AE The Netherlands

**Keywords:** Non-health outcomes, health promotion, economic evaluation, general population preferences, the Netherlands

## Abstract

**Background:**

Attention is increasing on the consideration of broader non-health outcomes in economic evaluations. It is unknown which non-health outcomes are valued as most relevant in the context of health promotion. The present study fills this gap by investigating the relative importance of non-health outcomes in a health promotion context.

**Method:**

We investigated the relative importance of ten non-health outcomes of health promotion programs not commonly captured in QALYs. Preferences were elicited from a sample of the Dutch general public (N = 549) by means of a ranking task. These preferences were analyzed using Borda scores and rank-ordered logit models.

**Results:**

The relative order of preference (from most to least important) was: self-confidence, insights into own (un)healthy behavior, perceived life control, knowledge about a certain health problem, social support, relaxation, better educational achievements, increased labor participation and work productivity, social participation, and a reduction in criminal behavior. The weight given to a particular non-health outcome was affected by the demographic variables age, gender, income, and education. Furthermore, in an open question, respondents mentioned a number of other relevant non-health outcomes, which we classified into outcomes relevant for the individual, the direct social environment, and for society as a whole.

**Conclusion:**

The study provides valuable insights in the non-health outcomes that are considered as most important by the Dutch general population. Ideally, researchers should include the most important non-health outcomes in economic evaluations of health promotion.

**Electronic supplementary material:**

The online version of this article (doi:10.1186/s12913-015-0908-y) contains supplementary material, which is available to authorized users.

## Background

Health promotion is defined by the World Health Organization (WHO) as the process of enabling people to increase control over and improve their health [1]. It involves a large variety of activities that move beyond individual behavior and contains a wide range of social and environmental interventions such as health education in schools, community development projects for disadvantaged mothers, the fluoridation of water, and campaigns to increase awareness of drink-driving and the dangers of smoking [2]. Current health promotion interventions tend to be complex, multi-factorial interventions which may take place on the individual, policy and physical environment levels [3]. As a consequence, health promotion programs may result in a wide variety of non-health outcomes like improvement of self-management capacities [4], reduced fear of falling [5], and increased health literacy [6]. Non-health outcomes like these are widely accepted as effect measures of health promotion programs. However, they are not captured by the narrow measures of health that are commonly used as outcome parameters in economic evaluations, such as life years gained, disease cases prevented or Quality Adjusted Life Years (QALYs). This may be due to the fact that the generic instruments used for the operationalization of QALYs, such as the EQ-5D and the SF-36, do not explicitly take into account outcomes that go beyond health.

The need to consider broader outcomes is increasingly acknowledged in the literature [3, 7–11]. However, there is only limited scientific work that provides insights into the type of non-health outcomes that could be (most) relevant in economic evaluations of health promotion (programs). Goebbels et al. [12], for example, used interviews and focus groups to explore what broader outcomes are particularly relevant from the perspective of participants in a lifestyle behavior change program, who were at risk for diabetes and cardiovascular disease. They identified non-health outcomes such as body satisfaction, stress reduction and relaxation, endurance, social interaction, feeling of control, overcoming addiction, feeling fresh and clean, and effort of making behavioral changes. Furthermore, using the intervention database of the Center for Healthy Living of the Dutch National Institute for Public Health and the Environment [13], Van Mastrigt et al. [14] identified a number of non-health outcomes in the area of health promotion and classified these outcomes based on their relevance to the individual, the direct social environment, and to society as a whole. The importance of non-health outcomes is also demonstrated empirically. For example, Borghi and Jan [15], using the contingent valuation method, found that in addition to their willingness to pay for health benefits, individuals were also willing to pay for some non-health benefits (e.g., knowledge sharing and increased confidence) of a group program to improve maternal and newborn health. Furthermore, Alayli-Goebbels et al. [16] demonstrated that both non-health as well as health outcomes significantly affected individuals’ preferences for lifestyle behavior change programs. Specifically, in a discrete choice experiment in which both health and non-health outcomes were included, they found that individuals valued health outcomes such as life expectancy and future health state value as well as non-health outcomes such as experienced control over lifestyle choices, days with sufficient relaxation, and body satisfaction. Despite the increasing attention and evidence for the importance of broader outcomes in the aforementioned literature, economic evaluations (of health promotion programs) hardly include any outcomes that go beyond health [17].

An explanation for this phenomenon may be associated with the available economic evaluation guidelines and, even more important, the related operationalization of the QALY. Most of the economic evaluation guidelines such as that of the International Society for Pharmacoeconomics and Outcomes Research (*ISPOR*) [18, 19], and the Dutch Health Care Insurance Board [20], are intended to be used for clinical studies and focus on measuring health (i.e., QALYs) as the main (or sometimes only) outcome measure of interest^1^.^1^ The QALY is a measure of health, representing life years adjusted for the experienced health-related quality of life. By using preferred instruments to determine health related quality of life, the quality adjustment factors are derived from changes in health states, and not from outcomes that go beyond health [21, 22]. This constitutes an important drawback for economic evaluations in the context of public health and health promotion. Interventions in this area usually generate non-health outcomes – at various operational levels [23]^2^^2^ – and are, despite exceptions such as smoking bans, more likely to lead to relatively minor or no improvements in terms of health in comparison to clinical interventions. Also, health improvements related to health promotion often occur in the far-away future. Although it may be possible, theoretically, to capture non-health benefits within the QALY framework [16, 24, 25] as yet there is no feasible and generally accepted method for this purpose. In the UK, the National Institute for Health and Care Excellence (NICE) established the Centre for Public Health Excellence [26]^3^^3^ and implemented new guidelines that clarified the necessity of using a range of methods for economic evaluations in the field of public health. Specifically, these guidelines suggested taking into account non-health outcomes by using a cost consequence approach (in which all costs and benefits are reported separately) or cost benefit approach (in which both costs and benefits are valued in monetary terms) [27, 28]. Note that, besides these approaches, cost-effectiveness analysis (in which health benefits are measured in natural units such as life years saved or number of days with disability) or cost utility analysis (in which benefits are measured via both the quantitative as well as qualitative aspects of health using an utility-based measure (e.g., quality of adjusted life years)) are also well-known economic evaluation methods [22]. However, regardless of the method used, the large scope of potentially relevant broader outcomes [12, 14] is likely to create a difficult task for researchers who need to decide what outcomes should be considered.

Despite its theoretical and practical relevance, there is only limited scientific work that provides insights into the type of non-health outcomes that are (most) relevant to consider for use in economic evaluations of health promotion (programs). We therefore build on this research stream [10–12, 14–16] by providing new insights regarding the relative importance of broader outcomes. Specifically, this study aims to investigate the relative importance of ten recognized non-health outcomes that are common across different areas of health promotion and to identify other potentially relevant broader outcomes of health promotion programs from the perspective of the Dutch open population. The most important non-health outcomes may be used by researchers for inclusion in economic evaluations of health promotion.

## Methods

### Sample

Respondents were recruited from a commercial consumer panel. The sample was selected to be representative of the Dutch population in terms of age, gender and education. We used a representative sample for the Dutch general public (N = 578) as the open population is a good source from which to derive the value of benefit [29]. The surveys were administered at different locations in the Netherlands (i.e., Utrecht, Maastricht, and Enschede) in the autumn of 2012. Respondents were asked to participate in our short (printed) survey after they had completed an interview in which they evaluated health states based on Euroqols’ EQ-5D dimensions on a computer [30]. For their participation in the combined interviews they received a financial incentive of 20 Euros and an additional 7.50 Euros for travel reimbursement. No other incentives were received for participating in our survey. This research was conducted according to the principles of the declaration of Helsinki. Ethical approval was not required because the survey aimed to explore opinions of the general population towards a topic of non-sensitive nature and is therefore exempted from ethical review under the scope of the Medical Research Involving Human Subjects Act (WMO), which protects the rights and safety of the study participants in the Netherlands [31].

### Questionnaire

The survey (Additional file 1: Appendix A) started with an explanation that in addition to health aspects, other aspects could play a role in health care in the context of health promotion. This was followed by a definition of health promotion. Then we presented respondents a ranking task in which they had to rank in order of importance ten different non-health outcomes in the context of health promotion. We emphasized that the ranking task was not about personal experiences, but more about how important respondents believed that the presented aspects were in general. Finally, respondents were asked to write down other (potentially missing) outcomes not mentioned in the ranking task in an open question.

### Selection of the non-health outcomes

The non-health outcomes were obtained from the study of Van Mastrigt et al. [14] that identified relevant broader outcomes in a health promotion context by searching the I-database of the Center for Healthy Living of the Dutch National Institute for Public Health and the Environment [13]. At the moment of consultation (February 2012), this database contained 2466 interventions, with a judgment of the level of evidence for effectiveness of the intervention. For the 23 interventions that were awarded the highest level of evidence (i.e., interventions for which there is at least one (quasi) experimental study with a follow-up period of six months or more), Van Mastrigt et al. listed 34 relevant non-health outcomes [14]. To achieve a manageable cognitive burden on respondents [32], we selected ten from these 34 non-health outcomes for inclusion in the ranking task. First, we classified the non-health outcomes into three non-health outcome categories (i.e., outcomes relevant for the individual, outcomes relevant for the direct social environment, and outcomes relevant for society as a whole). Then, we selected the ten most important outcomes based on their frequency of occurrence in the 23 interventions searched [14] with the prerequisite that there was at least one non-health outcome for each category. The final outcomes used in the survey were: knowledge about a certain health problem, insights into own (un)healthy behavior, self-confidence, relaxation, perceived life control, social support, better educational achievements, increased labor participation and work productivity, a reduction in criminal behavior, and social participation.

### Analysis

We investigated the data using Borda scores [33, 34] and rank-ordered logit regression models (STATA 12), following methods as explained by Dolan and Tsuchiya [35]. We calculated Borda scores – which represent aggregate ranks – to determine the relative importance of the non-health outcomes and reported the results as the proportion of respondents who rank a certain outcome in a specific sequence. In order to calculate Borda scores, we assumed that it is possible to treat the rank scores as cardinal data [35].

To confirm the relative importance results, as obtained from the Borda score method [33, 34], and to investigate whether particular subgroups of individuals had heterogeneous preferences for the non-health outcomes, we also estimated several rank-ordered logit models. First, we estimated a (base) model that contained only nine dummy coded variables as independent variables that each represented (the presence of) a particular non-health outcome from the ranking task (coded 1 if the outcome is “present” and 0 otherwise). The ranking of the non-health aspects was used as a dependent variable in all reported models. We predicted the probabilities of first rank for each non-health outcome, as the raw beta estimates in the rank-ordered logit model cannot be interpreted directly. These probabilities indicated the relative importance of the non-health outcomes and were used as a test for determining the performance of the rank-ordered logit models. More specifically, we compared the rank ordering of the predicted probabilities with the rank ordering based on the Borda scores, and calculated the correlation coefficient between the predicted probabilities and the Borda scores [35].

Second, we estimated several extended models that also included interactions of the non-health outcome dummies with the demographic characteristics age, gender, income, ethnicity, and education. The interactions provided information regarding the presence of preference heterogeneity (i.e., if particular subgroups of individuals, for example older people, attach more weight to a specific non-health outcome than do younger people). As we lacked power to estimate a model including all interactions at once, we estimated multiple models in which we included interactions of the outcomes with a single particular demographic characteristic (e.g., gender). We tested the performance of these alternative models by comparing their Log-Likelihood (LL) with the LL of the (base) model that included only the non-health outcome dummies.

Finally, we systematically classified respondents’ answers to the open question about the non-health outcomes that were also considered important in the following three non-health outcome categories; outcomes relevant for the individual, outcomes relevant for the direct social environment, and outcomes relevant for the society as a whole. The coding was performed by two researchers (GM and TB) and agreed upon in a consensus meeting (AAG, GM, SE, and TB).

## Results

### Respondents

From the 580 respondents that completed the earlier computer-based interview about the evaluation of health states [30], 578 respondents participated in our questionnaire (Enschede N = 202, Utrecht N = 184, and Maastricht N = 192). From these respondents, 557 (96.37 %) completed the ranking task successfully. Twenty-one respondents (3.63 %) either did not rank the outcomes at all (N = 1), ranked only part of the outcomes (N = 11), or gave different outcomes the same ranking (N = 9). We deleted the data of the 29 respondents who (a) did not complete the ranking task successfully (N = 21) or (b) who had missing details on demographic variables (N = 8). Finally, we used data of 549 respondents in the analysis, of which 254 were male (46.27 %) and 295 were female (53.73 %). The average respondent age was 47.5 years, ranging from 18 to 88 years. We refer to Table 1 for an overview of the other demographic characteristics of our sample – which is representative of the Dutch open population based on age, gender, and education in 2012 [36].

**Table 1:** Demographic characteristics of the sample

Demographic characteristic	
Age (average)	47.52 years
*Male*	46.27 %
*Female*	53.73 %
Ethnicity	
*Dutch*	87.98 %
*Non-Dutch*	12.02 %
Education level	
*Higher education*	29.69 %
*Other*	70.31 %
Income	
*<30.000 Euro*	59.56 %
*> = 30.000Euro*	40.44 %
Self-reported health (average)^a^	80.24

^a^Self-reported health was measured using the Visual Analogue Scale (0–100)

where 100 is perfect health

### Borda scores and rank-ordered logit model output

We present the Borda scores in Table 2. Self-confidence has the highest Borda score (7.57) and can therefore be regarded as the most important non-health outcome. Reduction in criminal behavior has the lowest score (3.09). Interestingly, the aforementioned last four non-health outcomes (i.e., better educational achievements, increased labor participation and work productivity, social participation, and a reduction in criminal behavior) clearly have lower Borda scores, ranging from 3.09 to 4.15, and are therefore of less importance in comparison with the first six outcomes that have Borda scores ranging from 6.36 to 7.57 (Table 2). However, within these two groups of more and less important non-health outcomes differences in relative importance are quite small. Within the group of more relevant non-health outcomes, the relative difference in Borda scores is only remarkably larger for the first outcome (self-confidence). Therefore, self-confidence can be regarded unambiguously as the most important non-health outcome in the (health promotion program) context of our study.

**Table 2:** Ranking results for the ten non-health outcomes

	Rank 1^b^	Rank 2	Rank 3	Rank 4	Rank 5	Rank 6	Rank 7	Rank 8	Rank 9	Rank 10	Borda score^d^	Predicted probability of first rank^c^
Self-confidence	**24.8 %**ª	18.8 %	16.0 %	14.2 %	7.1 %	7.3 %	4.4 %	3.3 %	2.4 %	1.8 %	7.57	0.21
Insights into own (un)healthy behavior	**18.2 %**	17.5 %	12.9 %	10.4 %	12.9 %	8.9 %	7.5 %	5.5 %	4.4 %	1.8 %	6.95	0.15
Perceived life control	11.8 %	15.1 %	16.0 %	**16.4 %**	13.7 %	11.1 %	5.8 %	5.8 %	2.2 %	2.0 %	6.82	0.15
Knowledge about a certain health problem	**15.7 %**	13.7 %	12.2 %	12.6 %	11.7 %	9.1 %	10.0 %	4.9 %	5.8 %	4.4 %	6.52	0.12
Social support	12.8 %	10.4 %	**15.1 %**	12.8 %	12.0 %	15.1 %	9.1 %	5.5 %	4.4 %	2.9 %	6.43	0.12
Relaxation	8.7 %	14.6 %	12.4 %	14.8 %	**15.5 %**	11.5 %	10.0 %	3.3 %	5.6 %	3.6 %	6.36	0.12
Better educational achievements	3.5 %	3.3 %	4.7 %	5.6 %	8.9 %	9.8 %	16.9 %	**18.8 %**	18.2 %	10.2 %	4.15	0.04
Increased labor participation and work prod.	0.9 %	3.1 %	3.6 %	6.0 %	7.8 %	8.9 %	16.2 %	**22.6 %**	19.1 %	11.7 %	3.82	0.04
Social participation	0.7 %	2.2 %	4.2 %	3.6 %	6.6 %	12.0 %	10.9 %	13.3 %	13.5 %	**33.0 %**	3.29	0.03
Reduction in criminal behavior	2.9 %	1.5 %	2.7 %	3.6 %	3.8 %	6.2 %	9.1 %	17.1 %	24.4 %	**28.6 %**	3.09	0.03

ªThe modes are in bold

^b^Rank 1 = most important, and rank 10 = least important

^c^The predicted probabilities are based on the (base) rank-ordered logit model

^d^The Borda scores indicate the relative order of preference for the non-health outcomes

Table 3 (model 1) shows the estimates of the base (rank-ordered logit) model. A comparison of the rank ordering of the predicted probabilities of first rank with the rank ordering based on the Borda scores (Table 2) shows a similar ranking of the non-health outcomes and thus indicates good model performance. The Pearson correlation coefficient between the predicted probabilities and the Borda scores is 0.97. In the models that include only the interactions for each demographic characteristic, we find significant interactions for a number of non-health outcomes with the demographic variables gender, age, income, and education (Additional file 2: Appendix B: models 2–6). It can thus be concluded that these specific groups of individuals value non-health outcomes differently and that there is preference heterogeneity. However, note that LL-ratio tests confirm an improvement in model fit only for the models in which the interactions of income (Additional file 2: Appendix B: model 4; *χ*^2^(9) = 29.9, p < 0.001) and education (Additional file 2: Appendix B: model 6; *χ*^2^(9) = 46.68, p < 0.001) are included. The interactions can be interpreted as follows: the positive (and significant) beta estimate for the interaction of gender (male) with increased labor participation and work productivity, for example, indicates that males consider the non-health outcome increased labor participation and work productivity more important than do females, *ceteris paribus*. We also performed additional analyses based on interactions of the non-health outcomes with self-reported health (visual analogue scale) to investigate if there is an effect of individuals’ health on the ranking of non-health outcomes. The results of these analyses (not reported) indicated that healthier individuals do not value non-health outcomes differently than less healthier individuals.

**Table 3:** Rank-ordered logit model results

	Model 1 (Base model)^a^	
	ß	Se
Knowledge about a certain health problem	1.49*	0.08
Insights into own (un)healthy behavior	1.74*	0.08
Self-confidence	2.08*	0.08
Relaxation	1.51*	0.08
Perceived life control	1.75*	0.08
Social support	1.51*	0.08
Better educational achievements	0.51*	0.07
Increased labor participation and work productivity	0.42*	0.07
Reduction in criminal behavior	−0.04	0.07
LR chi^2^	1777.88	
Df	(9)	
LL	−7403.38	

*Significant at p < 0.001

^a^The reference category is social participation

In total 277 (50.5 %) respondents provided an answer to the open question about potentially missing non-health outcomes. Reported outcomes that belong to the individual category, for example, were related to assertiveness, awareness of one’s place in society, creativity, norms and values, optimism/happiness, sexual performance, and view of life. Outcomes that belong to the direct social environment category, on the other hand, were related to the physical environment (housing) and social environment. Finally, outcomes in the societal category were related to the accessibility/affordability of health care, social economic situation, and therapy loyalty.

## Discussion

### Academic contributions

We explored the relative importance of non-health outcomes described in the literature and identified additional non-health outcomes relevant from the perspective of the general population in a health promotion context. The main result of our study is the derived relative importance of the ten selected non-health outcomes. Self-confidence, insights into own (un)healthy behavior, perceived life control, knowledge about a certain health problem, social support, and relaxation are the most important non-health outcomes of our study. These outcomes all belong to the individual category. Worth noting is that the first five of these non-health outcomes can be regarded as cognitions/beliefs. According to Prenger et al. [10] cognitive states have the potential to account for partial behavior change and should therefore be incorporated in economic evaluations .

The relative importance results are, to some extent, in line with the literature. The non-health outcomes knowledge sharing and increased confidence, from Borghi and Jan [15], and experienced control over lifestyle choices, days with sufficient relaxation, and body satisfaction used by Alayli-Goebbels et al. [16], were among the six most important outcomes in our study – although their descriptions were not entirely identical. More specifically, the non-health outcomes knowledge sharing and increased confidence from Borghi and Jan [15] are very similar to the outcomes knowledge about a certain health problem and self-confidence. In our study these outcomes were, respectively, the fourth and first most important non-health outcomes. The outcomes experienced control over lifestyle choices, days with sufficient relaxation, and body satisfaction, from Alayli-Goebbels et al. [16], are related to the outcomes perceived life control, relaxation, and (perhaps) self-confidence. In the present study, these outcomes were ranked, respectively, as the third, sixth and first most important ones. Despite the similar high relative importance of these outcomes, the willingness to pay (WTP) calculations in Alayli-Goebbels et al. [16] indicated that individuals were, on average, willing to pay more for additional days of relaxation than for increased experienced control and body satisfaction [16], while our study results indicated that control and self-confidence were relatively more important than relaxation.

Results of the present study suggest that the relative importance of the non-health outcomes used is affected by demographic background factors such as gender, age, education, and income. This indicates that it makes sense to consider what non-health outcomes can best be included in economic evaluations for health promotion programs that are meant for particular subgroups of individuals (e.g., females or older individuals). The finding that individuals have heterogeneous preferences for non-health outcomes was also confirmed in several recent studies that used discrete choice experiments [16, 25].

Finally, we identified other potentially relevant non-health outcomes within the following three non-health outcome categories: (1) outcomes relevant for the individual, (2) outcomes relevant for the direct social environment, and (3) outcomes relevant for society as a whole. Interestingly, a number of broader outcomes identified in the present study were not found in the search of Van Mastrigt et al. [14] such as creativity, optimism/happiness/ability to keep things in perspective, norms and values (respect), sexual performance, accessibility/affordability of health care, financial aspect/social economic situation, and therapy loyalty. The fact that non-health outcomes such as increased creativity and happiness were not identified in the searched interventions [14] could be an indication that health care promoters are insufficiently aware that individuals also value non-health outcomes like these. Initiatives such as the calculation of a happiness index could be useful in this context – as happiness and health may be related, but are not necessarily the same [37]. However, the specific character of the procedure used to select the non-health outcomes may also be a possible explanation that these outcomes were not identified.

### Limitations and directions for future research

Several limitations of the present study are worth mentioning. First, we included only non-health outcomes in the ranking task and respondents were not confronted with a task consisting of both health as well as non-health outcomes. A ranking task that includes both outcome types would make a direct comparison between these different outcomes possible, and might provide additional interesting information for researchers that have to decide about what outcomes to consider for use in economic evaluations in a health promotion context. Future research may therefore include both health as well as broader outcomes in a ranking-based study.

A second limitation is related to the limited number of non-health outcomes included in the survey and the procedure used to select these outcomes. Including more non-health outcomes would make the ranking task too complex and burdensome for respondents [32]. We therefore made a selection of ten non-health outcomes. However, the wide range of responses to the open question indicated that, besides the ten non-health outcomes addressed in our survey, other relevant outcomes resulting from health promotion programs could have been included in the ranking task as well. These outcomes might be the first to take into consideration in future studies that further investigate the (relative) importance of non-health outcomes in a health promotion context by using a blocked attribute design that allows researchers to include more attributes [38]. Furthermore, we selected the non-health outcomes based on frequency of occurrence in the published literature. Although we believe this reflects both participants of health promotion programs as well as economists most preferred non-health outcomes, some bias may have occurred as our method was not validated. The fact that the order of presenting the non-health outcomes was the same for each respondent may also have led to a slight preference of outcomes that were presented first. To avoid these potential biases, future studies may use focus groups to determine more precisely what non-health outcomes can best be included in ranking tasks and use different survey versions in which the order of presenting the non-health outcomes changes over respondents.

Third, respondents started the ranking task after they had completed an earlier valuation study concerning EQ-5D health states [30]. It may therefore be possible that they were already tired due to the completion of this study. A related issue is that the earlier study had an individual perspective (i.e., respondents’ were asked to indicate how *they* would evaluate health states). This may have prompted them to also complete our survey from this perspective. We therefore emphasized clearly that the ranking task was not about respondents’ personal experiences, but about how important the aspects were in general at the start of the survey. The finding that the most important non-health outcomes are all outcomes that belong to the individual category could be an indication that respondents acted from an individual perspective. However, it seems most likely that they simply perceived these outcomes as the most important ones given that they are found to be of high importance in earlier studies too [15, 16]. The fact that respondents started our survey after completing the earlier valuation study can also be seen as an advantage as it may have provided them with better insights about what outcomes not to consider when completing the survey.

Fourth, a substantial part of the reported answers to the open question were process-related (i.e., how a certain outcome could be obtained). Moreover, some respondents used the open question to comment on general real-world issues and mentioned health outcomes as an answer. However, overall, we had the impression that the respondents understood the open question and the ranking task quite well.

A final limitation may be that the analysis is based on a sample of the Dutch population only and that our findings therefore cannot be generalized over countries due to cultural differences that may lead to other results.

## Conclusion

Notwithstanding the aforementioned limitations this is the first study that examined the relative importance of several non-health outcomes that are common across different areas of health promotion. The results from our study offer interesting insights for the development of questionnaires related to the measurement of non-health outcomes. They also are a useful first step for researchers towards a better understanding of the relative importance of non-health outcomes of health promotion programs that focus, for example, on smoking cessation, stimulating healthy eating and exercising, and helping individuals with psychological problems. Ideally, the resulting most important non-health outcomes should be included in future economic evaluations of health promotion. A researcher who is performing an economic evaluation, and has instruments available that measure “self-confidence” and “insights into one’s unhealthy behavior”, for example, could use valuations of these outcomes in a cost utility analysis, as has been done with capabilities and the ICECAP instrument [39] – see also the article of Prenger et al. [40] that describes how to include self efficacy in economic evaluations. Another possibility would be to incorporate the non-health outcomes into a cost consequence analysis.

Investigating the importance of non-health outcomes in comparison with health outcomes and disentangling the existing relationships between the different possible non-health outcomes are interesting directions for further research in health promotion. The present work and further research in this domain of health care is particularly relevant given its rapidly increasing economic stakes [41].

## Additional files

Additional file 1:
**Appendix A.** Survey instrument (translated from Dutch). Additional file 2:
**Appendix B.** Rank-ordered logit model estimates with interactions per demographic characteristic.

## Footnotes

1 The focus on QALYs may also be a reason why measures of wellbeing that take broader outcomes into account are not used in real-world economic evaluations yet [39].

2 Public health interventions have a more diffused focus than clinical interventions and therefore work at different operational levels such as that of the community, population and society [23]. This diffused focus makes it difficult to calculate the cost-effectiveness of public health interventions.

3 The development of the Centre for Public Health Excellence followed after publication of the Wanless reports which pointed out that applying economic evaluation methods to multifaceted public health interventions is difficult [42, 43].
