# Supplementary material for: Exploring Outcomes to Consider in Economic Evaluations of Health Promotion Programs: What Broader Non-Health Outcomes Matter Most?
Source: BMC Health Serv Res. 2015 Jul 14;15:266. doi: 10.1186/s12913-015-0908-y (PMC4501101; doi:10.1186/s12913-015-0908-y)
Supplement: Additional file 1: — Appendix A. Survey instrument (translated from Dutch). [file 12913_2015_908_MOESM1_ESM.docx]

**Appendix A: Survey instrument (translated from Dutch)**

Dear respondent,

You just answered questions on views about health and illness behind the computer. In these questions attention was given to several aspects of health, namely:

- Mobility (problems in walking)
- Self-care (problems washing or dressing)
- Usual activities (problems with work, study, housework, family or leisure activities)
- Pain/discomfort
- Anxiety/depression

In relation to health also **other aspects** can play a role. This short questionnaire is about how important you think these other aspects are, particularly in the field of **health promotion**. Your answers from this questionnaire will also be used anonymously. To link the data to questions you answered before we kindly ask you to write down your birth date below.

**Birth date** ………………………………

We are going to ask you about **other aspects** that play a role in **health promotion**.

**Health promotion** aims to adjust the environment and lifestyle of individuals in such a way that it positively influences their health. For example, consider programs about smoking cessation, stimulating healthy eating and exercising, and helping individuals with psychological problems.

Besides improved health, these programs may also have other outcomes that are not directly health- related. Examples of such outcomes are described in the table on the next page.

**QUESTION 1**

**Could you indicate how important these outcomes are by ranking them in terms of importance in the first column?**

The number 10 belongs to the aspect that you find most important and the number 1 belongs to the least important aspect*. Please also rank all aspects in between, from most important (highest number) to least important (lowest number). Note again that this task is about aspects that are relevant in health promotion.

The ranking task is **not** about your personal experiences with these aspects, but about how important you think these aspects are **IN GENERAL**.

**Aspect besides improved health**

| **Importance** | **10 = most important, 1 = least important; please also rank 2-9 in terms of importance** |
| --- | --- |
|  | **Knowledge about a certain health problem**  Health promotion is usually aimed at providing information. For example: It enables you to know more about the possibilities of handling a depression or preventing diabetes. |
|  | **Insights into own (un)healthy behavior**  More insights into your own (un)healthy behavior and pitfalls may help you to change your behavior. For example: If someone has a drinking problem, insights into one’s unhealthy behavior can be helpful in avoiding situations that seduce one to drink too much alcohol. |
|  | **Self-confidence**  Greater self-confidence means that you believe more in yourself and that you have confidence in your own strengths and skills irrespective of the situation you are in. For example: A program to prevent falling gives elderly people with fear of falling more confidence to go outside. |
|  | **Relaxation**  More relaxation means that you are able to relax more and free your mind. For example: A higher degree of relaxation may be achieved by a course that teaches skills in dealing with stress. |
|  | **Perceived life control**  For example: An improvement in life control may take place when people with social fear have more control of their situation and can communicate more easily with other people. Another example of more control is that people who are overweight are better able to make healthy lifestyle choices. |
|  | **Social support**  Social support is related to the degree of advice, appreciation, practical help and support you get from individuals in your environment such as family and friends. For example: If your partner encourages you, this can help you to eat more healthily or to exercise more. |
|  | **Better educational achievements**  Anti-bullying campaigns at schools may improve concentration in children during class and may help them to achieve higher grades. |
|  | **Increased labor participation and work productivity**  For example: Participation in a course for the prevention of panic disorders may help someone to be better able to carry out his work. |
|  | **A reduction in criminal behavior**  For example: By taking part in an alcohol prevention program someone can become less aggressive, which may lead to a reduced involvement in fights. |
|  | **Social participation**  For example: Participation in a neighborhood exercise program may help women of ethnic minorities in making contacts with non-foreign citizens and become part of the community. |

***** In the results of this article the most important attribute (score 10 in the survey) is indicated by rank 1.
