# Supplementary material for: Exploring Outcomes to Consider in Economic Evaluations of Health Promotion Programs: What Broader Non-Health Outcomes Matter Most?
Source: BMC Health Serv Res. 2015 Jul 14;15:266. doi: 10.1186/s12913-015-0908-y (PMC4501101; doi:10.1186/s12913-015-0908-y)
Supplement: Additional file 2: — Appendix B. Rank-ordered logit model estimates with interactions per demographic characteristic. [file 12913_2015_908_MOESM2_ESM.docx]

**Appendix B: Rank-ordered logit model estimates with interactions per demographic characteristic**

|  | Model 1^b^  (Base) | | Model 2  (Gender; male = 1) | | Model 3  (Age; continuous) | | Model 4  (Income;  <30.000 = 1) | | Model 5  (Ethnicity; autochthonous = 1) | | Model 6  (Education; Univ. of professional education or univ. of science = 1) | |
| --- | --- | --- | --- | --- | --- | --- | --- | --- | --- | --- | --- | --- |
|  | ß | Se | ß | Se | ß | Se | ß | Se | ß | Se | ß | Se |
| Knowledge about a certain health problem | 1.49* | 0.08 | 1.50* | 0.10 | 1.19* | 0.25 | 1.76* | 0.12 | 1.31* | 0.22 | 1.64* | 0.09 |
| Insights into own (un)healthy behavior | 1.74* | 0.08 | 1.60* | 0.10 | 1.52* | 0.25 | 2.12* | 0.13 | 1.41* | 0.22 | 1.78* | 0.09 |
| Self-confidence | 2.08* | 0.08 | 2.00* | 0.11 | 1.40* | 0.25 | 2.35* | 0.13 | 1.86* | 0.22 | 2.24* | 0.10 |
| Relaxation | 1.51* | 0.08 | 1.46* | 0.10 | 0.98* | 0.24 | 1.61* | 0.12 | 1.27* | 0.21 | 1.72* | 0.09 |
| Perceived life control | 1.75* | 0.08 | 1.71* | 0.10 | 1.61* | 0.25 | 2.03* | 0.12 | 1.60* | 0.22 | 1.85* | 0.09 |
| Social support | 1.51* | 0.08 | 1.46* | 0.10 | 1.43* | 0.25 | 1.55* | 0.12 | 1.22* | 0.21 | 1.66* | 0.09 |
| Better educational achievements | 0.51* | 0.07 | 0.52* | 0.10 | 0.33 | 0.24 | 0.68* | 0.12 | 0.53*** | 0.21 | 0.70* | 0.09 |
| Increased labor participation and work productivity | 0.42* | 0.07 | 0.28** | 0.10 | 0.13 | 0.24 | 0.62* | 0.12 | 0.16 | 0.21 | 0.49* | 0.09 |
| Reduction in criminal behavior | -0.04 | 0.07 | -0.12 | 0.10 | -0.31 | 0.25 | 0.06 | 0.12 | 0.02 | 0.21 | 0.19*** | 0.09 |
|  |  |  |  |  |  |  |  |  |  |  |  |  |
| Variable*Knowledge about a certain health problem^a^ |  |  | -0.00 | 0.15 | 0.01 | 0.01 | -0.45** | 0.16 | 0.21 | 0.23 | -0.47** | 0.16 |
| Variable*Insights into own (un)healthy behavior |  |  | 0.31*** | 0.15 | 0.00 | 0.01 | -0.62* | 0.16 | 0.38 | 0.23 | -0.11 | 0.17 |
| Variable*Self-confidence |  |  | 0.17 | 0.16 | 0.01** | 0.01 | -0.44** | 0.16 | 0.25 | 0.24 | -0.52** | 0.17 |
| Variable*Relaxation |  |  | 0.11 | 0.15 | 0.01*** | 0.00 | -0.16 | 0.16 | 0.27 | 0.23 | -0.69* | 0.16 |
| Variable*Perceived life control |  |  | 0.11 | 0.15 | 0.00 | 0.01 | -0.44** | 0.16 | 0.17 | 0.23 | -0.30 | 0.17 |
| Variable*Social support |  |  | 0.10 | 0.15 | 0.00 | 0.01 | -0.06 | 0.16 | 0.33 | 0.23 | -0.48** | 0.16 |
| Variable*Better educational achievements |  |  | -0.01 | 0.15 | 0.00 | 0.00 | -0.28 | 0.15 | -0.02 | 0.22 | -0.62* | 0.16 |
| Variable*Increased labor participation and work prod. |  |  | 0.31*** | 0.15 | 0.01 | 0.00 | -0.35*** | 0.15 | 0.29 | 0.22 | -0.26 | 0.16 |
| Variable*Reduction in criminal behavior |  |  | 0.17 | 0.15 | 0.01 | 0.00 | -0.17 | 0.15 | -0.07 | 0.23 | -0.80* | 0.17 |
|  |  |  |  |  |  |  |  |  |  |  |  |  |
| LR chi²  Df  LL  -2LL ratio test ^c^ | 1777.88  (9)  -7403.38 | | 1790.60  (18)  -7397.02  χ²(9)=12.72 | | 1793.64  (18)  -7395.50  χ²(9)=15.76 | | 1807.79  (18)  -7388.43  χ²(9)=29.9* | | 1786.07  (18)  -7399.29  χ²(9)=8.18 | | 1824.57  (18)  -7380.04  χ²(9)=46.68* | |

* Significant at p < 0.001, ** Significant at p < 0.01, *** Significant at p < 0.05.

^a^ “Variable” means the particular demographic background variable used as interaction in the model (e.g., in model 2 “Variable * Knowledge…” should be read as “Gender * Knowledge…”).

^b^ The reference category is social participation (in all reported models).

^c^ -2LL-ratio test for current model relative to the base model.
